# Lysosomal transmembrane protein 5: Impact on immune cell function and implications for immune-related deficiencies

**DOI:** 10.1016/j.heliyon.2024.e36705

**Published:** 2024-08-22

**Authors:** Peng-Peng Sun, Shi-Xia Liao, Peng Sang, Mao-Mao Liu, Ji-Bin Yang

**Affiliations:** a Department of Orthopedics Surgery, Affiliated Hospital of Zunyi Medical University, Zunyi, China; b Department of Respiratory and Critical Care Medicine, Affiliated Hospital of Zunyi Medical University, Zunyi, China; c Department of Anesthesiology, West China Hospital, Sichuan University, Chengdu, Sichuan, China

**Keywords:** LAPTM5, Immunity, Immune infiltration, Solid tumor, Non-solid tumors, Potential target

## Abstract

Lysosomal transmembrane protein 5 (LAPTM5) is a lysosomal-associated protein that interacts with surface receptors on various immune cells, including B cells, T cells, macrophages and dendritic cells. Dysregulated expression of LAPTM5 is implicated in the development of multiple immune system-related diseases. In the context of tumors, elevated LAPTM5 levels in immune cells are associated with decreased cell membrane levels of T cell receptors (TCR) or B cell receptors (BCR), leading to impaired antigen presentation and immune escape, thereby promoting tumor progression. Besides, LAPTM5 is critical for inducing non-apoptotic cell death in tumor parenchymal cells since its downregulation leads to inhibition of the cell death pathway in the tumor parenchyma and subsequent enhanced tumorigenesis. LAPTM5 also affects the cell cycle as the elevated LAPTM5 expression in solid tumors causes its inability to block the G0/G1 stage. In non-solid tumors, abnormal LAPTM5 expression disrupts blood cell development and causes irregular proliferation. Furthermore, in the nervous system, aberrant LAPTM5 expression in microglia is correlated with Alzheimer's disease severity. In this context, further preclinical research is essential to validate LAPTM5 as a potential target for diagnosis, therapy, and prognosis in immune-related disorders and tumors. This review summarized the current insights into LAPTM5's role in tumors and immune-related deficits, highlighting its potential as a valuable biomarker and therapeutic target.

## 1 The basic structure of LAPTM5

Lysosomal Protein Transmembrane 5 (LAPTM5) is a 30 kDa lysosomal protein featuring five transmembrane fragments, with its N terminus located within the lysosome and its C terminus located within the cytoplasm. LAPTM5 contains three PY motifs (L/PPxY) that bind to the Nedd4-WW domain, and a ubiquitin interaction motif (UIM). The Nedd4-LAPTM5 complex recruits ubiquitinated GGA3, and the binding of GGA3 to LAPTM5-UIM promotes the transfer of LAPTM5 from the Golgi apparatus to the lysosome, an event independent of LAPTM5 ubiquitination. When Nedd4 or GGA3 is knocked down, or when the PY or UIM motif is mutated, LAPTM5 is retained in the Golgi apparatus [1].

## 2 The physiologic role and function of LAPTM5

LAPTM5 is encoded by the LAPTM5 gene, also known as the E3 protein. During embryonic development, the LAPTM5 gene is expressed in both hematopoietic and non-hematopoietic tissues [2]. Within the cell lineage, the gene is specifically expressed in the hematopoietic lineage, whereas in normal adult tissues, its mRNA is predomidantly detected at high levels in both lymphoid and myeloid tissues. The expression pattern of the gene, coupled with the preliminary evidence revealing the interaction between LAPTM5 protein and ubiquitin suggests that this protein may play crucial roles in embryogenesis and in adult hematopoietic cells function [[1], [2], [3]]. Moreover, LAPTM5 expression levels were associated with the distribution and number of B cell receptors (BCR), T cell receptors (TCR), and dendritic cell receptors, underscoring its significant involvement in immune system-related diseases [4]. Additional evidence demonstrates that LAPTM5 is differentially expressed in various tumor tissues, indicating its potential as a valuable marker for the diagnosis, treatment, and prognostic evaluation of tumors [[5], [6], [7], [8], [9], [10], [11], [12], [13], [14], [15], [16], [17], [18], [19], [20]]. In this review, we provide a succinct summary of current research findings that elucidate the critical roles of LAPTM5 in tumors and immune-related diseases. These insights might offer valuable directions for further studies.

## 3 Role of LAPTM5 in the immune system

LAPTM5 is a 30-kDa protein predominantly expressed in lymphoid and myeloid cells [2]. LAPTM5plays a crucial role in regulating multiple pathways within immune cells. Previous studies have revealed that LAPTM5 suppresses excessive T cell activation by down-modulating surface TCR levels [21]. Additionally, it has been shown that LAPTM5 negatively regulates surface BCR levels on mature B cells, with LAPTM5 deficiency resulting in enhanced B cell activation and autoantibody production [22].

### 3.1 Function of LAPTM5 in T cells

**Fig. 1:**
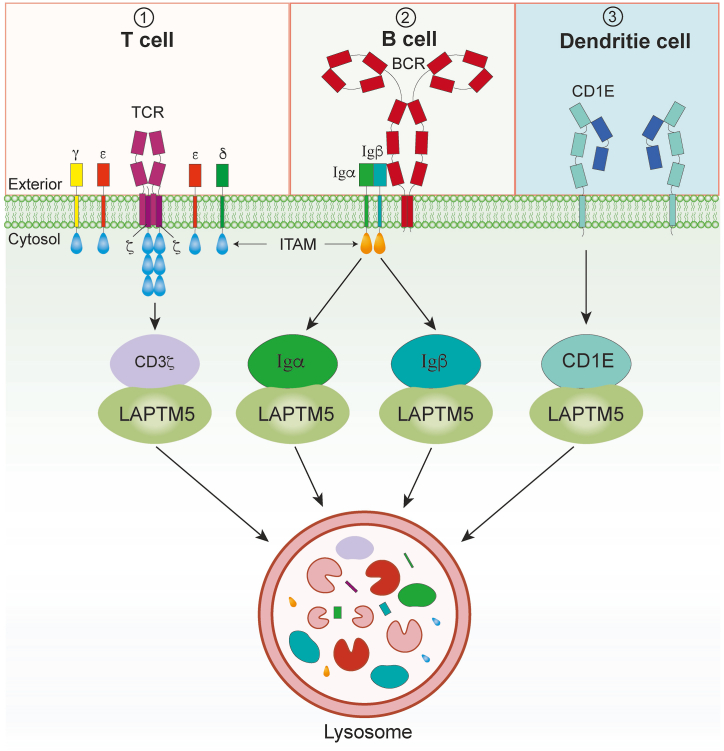
The main function of LAPTM5 in immune cells. LAPTM5 plays a crucial role in regulating receptor levels within immune cells. Induction of LAPTM5 facilitates the translocation of a significant number of intracellular pre-BCR complexes to the lysosomal compartment, which results in their lysosomal degradation and subsequently downregulating surface pre-BCR levels. LAPTM5 interacting with the BCR or TCR complexes can promote their degradation in the lysosomal compartment. The transmembrane precursor of CD1e proteins is transported to lysosomes, where it is cleaved into an active soluble form. LAPTM5 colocalizes with CD1e in the trans-Golgi and late endosomal compartments, further highlighting its role in receptor trafficking and processing. TCR, T cell receptor; BCR, B cell receptor.

TCR undergoes constitutive internalization, and is then either recycled back to the plasma membrane or sorted to lysosomes for degradation [23,24]. Modifications in these processes can affect steady-state TCR expression on the cell surface [[24], [25], [26]]. Following TCR triggering, a similar process occurs, but with much faster kinetics [27]. In double-positive (DP) thymocytes, SLAP and c-Cbl promote the degradation of the internalized TCR [28]. Both SLAP and c-Cbl co-target CD3 for ubiquitination and degradation, thus preventing the accumulation of the fully assembled recycling TCR [29,30]. Deficiency in SLAP and/or c-Cbl resulted in elevated surface TCR expression on DP thymocytes and enhanced positive selection of thymocytes expressing class MHC II-restricted TCR, which resulted in sustained TCR signaling [31,32]. LAPTM5 interacts with CD3***ζ*** through its PY3 and UIM motif to promote CD3***ζ*** degradation, thereby downregulating TCR (Fig. 1). In the case of LAPTM5 knockdown, the proliferation of splenic T cells upon CD3 stimulation was continuously increased, along with the IL-2 and IFN-γ production [21]. However, LAPTM5 specifically affects TCR expression in c-Cbl or SLAP-deficient CD4^+^CD8^+^ DP thymocytes, but not in single-positive (SP) thymocytes. This indicates that LAPTM5 deletion specifically impacts CD3***ζ*** protein expression in DP phase of thymocyte differentiation [21]. Another study showed that LAPTM5 can promote lysosomal degradation of CD3 in the cytoplasm, rather than at the cell membrane [33].

### 3.2 Function of LAPTM5 in B cells

B cell development in the bone marrow is characterized by the sequential production of immunoglobulin (Ig) heavy (H) and light (L) chains [34,35]. At the pre-B cell stage, the L chain is not yet produced, and the H chain is covalently linked to the substitution L (SL) chains composed of VpreB and ***λ***5. This complex is noncovalently associated with the signal-transducing Ig***α***-Ig***β*** heterodimer to form the pre-B cell receptor (BCR) [36]. Unlike mature BCR, pre-BCR does not act as a receptor for antigen recognition, but conversely plays a crucial role in an antigen-independent manner in B cell development within the fetal liver and adult bone marrow. Dysregulation of pre-BCR expression and signaling leads to the impaired B-cell development, the leukemogenesis of pre-B cells, and the production of autoantibodies [37]. Induction of LAPTM5 does not significantly affect the internalization of pre-BCR on the cell surface. However, it promotes the translocation of a substantial number of intracellular pre-BCR complexes to the lysosomal compartment, resulting their lysosomal degradation and a subsequent decrease in the surface pre-BCR levels [38] (Fig. 1). Furthermore, LAPTM5 interacts with the BCR complex and promote its degradation in the lysosomal compartment. This LAPTM5-mediated downregulation of BCR is an important mechanism for preventing B cell hyperactivation and autoantibody production. In a recent study, antigen-specific B cells were increased in LAPTM5-deficient mice, leading to elevating antibodies production. With these mice age, their serum IgM and autoantibody titers increase, resulting in immune complexes deposition in the kidney [22]. These results suggest a critical role for LAPTM5 in the negative regulation of surface BCR levels and B cell activation [4].

### 3.3 Role of LAPTM5 in macrophages

LAPTM5 expression is crucial for pro-inflammatory cytokine secretion in response to Toll-like receptor ligands. Previous studies reported that macrophages in LAPTM5^−/−^mice exhibited reduced activation of NF-kB and mitogen-activated protein kinase (MAPK) signaling, which are mediated by tumor necrosis factor (TNF) receptors and multiple pattern recognition receptors across different cell types [39]. This reduction in signaling is associated with decreased ubiquitination of Receptor Interaction Protein 1 (RIP1) following TNF stimulation in LAPTM5^−/−^ macrophages. Additionally, macrophages derived from LAPTM5^−/−^ mice showed an upregulation of A20 levels, a ubiquitin-editing enzyme responsible for the deubiquitination of RIP1 and the subsequent NF-kB activation [39]. These findings suggest that, in contrast to its negative role in T and B cell activation, LAPTM5 acts as a positive modulator of inflammatory signaling pathways, promoting cytokine secretion in macrophages. Interestingly, it has been shown that the HIV-1 accessory proteins Vif, Vpu, and Nef enhance infection by overcoming the inhibitory effects of the host cell restriction factors APOBEC3G, Tetherin, and SERINC5, respectively [[40], [41], [42], [43]]. HIV-1 infection is enhanced when LAPTM5 is inhibited by Vpr in macrophages, while Vpr does not enhance HIV-1 infection in the absence of LAPTM5 [44]. LAPTM5 facilitates the transport of HIV-1 envelope glycoproteins to lysosomes for degradation, thereby inhibiting virion infectivity. Vpr counteracts its limiting effect by triggering LAPTM5 degradation via DCAF1. Notably, LAPTM5 is highly expressed in macrophages, but not in CD4^+^ T lymphocytes. In primary CD4^+^ T cells, the re-expression of LAPTM5 contributes to a Vpr-dependent promotion of HIV-1 infection, as was observed in macrophages [44].

### 3.4 Role of LAPTM5 in dendritic cells

The CD1e proteins are involved in the presentation of lipid antigens in DCs. Their transmembrane precursor is transported to lysosomes, where it is cleaved into an active soluble form. LAPTM5, as a molecular partner, colocalizes with CD1e in the trans-Golgi and late endosomal compartments (Fig. 1). The number of LAPTM5/CD1e complexes increases when cells are treated with bafilomycin, possibly due to LAPTM5 avoiding the effects of lysosomal proteases [45]. Previously, LAPTM5 has been shown to act as a platform that recruits ubiquitin ligases and promotes receptor trafficking to lysosomes. The interaction of LAPTM5 with CD1e and its co-localization in the antigen-processing compartments suggest that LAPTM5 may influence the role of CD1e in lipid-antigen presentation.

## 4 The role of LAPTM5 in tumors

Lysosomes play a vital role in maintaining cellular homeostasis and contribute to various cancer hallmarks, making them an attracting target for cancer treatment. Dysregulated lysosomal function, such as alterations in lysosomal composition, volume, cellular distribution, and enzyme activity, promotes cancer cell growth and survival [46]. LAPTM5, a protein preferentially expressed in immune cells, interacts with the Nedd4 family of ubiquitin ligases. LAPTM5 functions as a negative regulator of T and B cell receptor levels at the plasma membrane, while also serving as a positive modulator of inflammatory signaling pathways, thereby enhancing cytokine secretion in macrophages. Several studies have linked LAPTM5 to tumor development, with some suggesting that LAPTM5 functions as a tumor suppressor, while others argue that it may act as an oncoprotein [10,47]. Specifically, LAPTM5 knockdown have suppressed ovarian cell metastasis and inhibited tumorigenesis through modulating MAPK and TGFβ/Smad signaling pathways [48]. However, controversial involvement of LAPTM5 extends to the hematopoietic system. For instance, LAPTM5 might function as a tumor-suppressive protein in human multiple myeloma [15], yet it could also promote tumor growth in human B-cell lymphomas [13,49]. Consequently, the role of LAPTM5 in the pathogenesis of malignancies remains complicated.

### 4.1 The role of LAPTM5 in glioma and neuroblastoma

LAPTM5 has shown contradictory outcomes in gliomas. One of related studies showed that high protein levels of LAPTM5 were associated with a poor survival rate in glioma patients [50]. Conversely, an *in vivo* study screening genes involved in glioma invasion identified LAPTM5 as a highly anti-invasive gene for glioblastoma. In that study, LAPTM5 expression was downregulated in the invasive anterior portion of the U87 MG-derived in situ implanted tumors. In addition, methylation analysis revealed that LAPTM5 expression is negatively regulated by methylation at the CpG sites, Cg10001720 and Cg12732155. LAPTM5 knockdown resulted in CD40-mediated NF-B activation, which enhanced invasion, clonality, and temozolomide resistance in glioma. Furthermore, LAPTM5 expression was correlated with a better overall survival in glioblastoma patients, contingent on the CD40 expression status. Specifically, LAPTM5 exhibits tumor suppression and sensitivity to temozolomide by inhibiting the CD40-mediated NF-kB activation in CD40-positive gliomas [5]. Therefore, LAPTM5 may present as a potential biomarker for the sensitivity of CD40-positive glioblastoma to temozolomide.

**Fig. 2:**
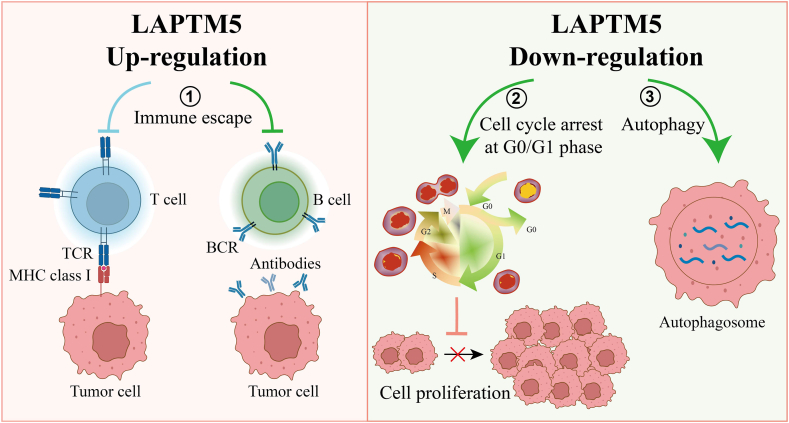
Major functions of LAPTM5 in tumor diseases. Accumulation of LAPTM5 protein is critical for induction of non-apoptotic cell death. LAPTM5 knockdown triggers a cell-cycle arrest in the G0/G1 phase and results in delayed tumor cell growth, and inhibition of migration and invasion. LAPTM5 knockdown can lead to enhanced autophagy, which may contribute to non-apoptotic cell death pathways. BCR, B cell receptor; TCR, T cell receptor; Up, upregulation of LAPTM5; Down, downregulation of LAPTM5.

Neuroblastoma, the most common solid tumor in children, is characterized by heterogeneous clinical behavior. LAPTM5 expression is generally downregulated in neuroblastoma cells due to cell-specific DNA methylation, but is upregulated in degenerated neuroblastoma cells in locally degenerated tumors regions, as detected by large-scale screening [12]. *In vitro* experiments have demonstrated that expression recovery and accumulation of LAPTM5 protein are critical for the induction of non-apoptotic cell death [12] (Fig. 2). LAPTM5 protein levels are negatively regulated by the E3 ubiquitin ligase ITCH, which directly binds to the PPxY motif of LAPTM5 through its WW domain and promotes ubiquitination through its HECT-type ligase domain [47]. Overexpression of ITCH leads to the degradation of the LAPTM5 protein, while inhibition of ITCH enhances LAPTM5 accumulation in neuroblastoma cells, leading to increased cell death [47]. The therapeutic potential of LAPTM5 appears significant and warrants further in-depth investigations to validate its role as a therapeutic target in neuroblastoma.

### 4.2 The role of LAPTM5 in liver cancer

Chronic hepatitis, cirrhosis, and liver cancer are interconnected stages in the progression of liver disease [51,52]. Patients suffering from chronic hepatitis, long-term alcohol abuse, and autoimmune diseases frequently develop liver fibrosis [[53], [54], [55], [56]]. A study using a fibrosis model of the multicellular liver microtissues (MTs) caused by TGF-β1 suggests that LAPTM5 may be a novel cell-specific fibrosis marker [57]. Alcoholic liver disease (ALD) is characterized by extensive deposition of extracellular matrix proteins and fibrous scarring. Hepatic stellate cells (HSCs) are the main source of collagen type 1 in ALD-associated fibrosis. Notably, RNA sequencing analysis have identified uniquely upregulated genes in HSCs of ALD patients, with LAPTP5 included [58]. This observation indicates that aberrant LAPTM5 expression may occur early in tumorigenesis or even during phases of tissue damage that predispose the liver to tumor development.

### 4.3 Role of LAPTM5 in other solid tumors

LAPTM5 expression has been significantly upregulated in various solid tumors. Data from the Cancer Genome Atlas (TCGA) showed that LAPTM5 is significantly upregulated in clear-cell renal cell carcinoma (ccRCC), and uncovered its positive correlation with the overall survival rates in patients [59]. As known, lung cancer poses significant public health challenges due to its high morbidity and mortality rates, which has also shown abnormal methylation and expression profiles of LAPTM5 [60,61]. The gene expression profiles and DNA methylation profiles illustrated that LAPTM5 is abnormally methylated in lung cancer [62], with its methylation status correlated with the tumor differentiation status [19]. TCGA data on lung squamous cell lung cancer (LUSC) indicated that LAPTM5 expression is positively associated with the infiltration of M1 and M2 macrophages, resting mast cells and CD8^+^ T cells, and negatively associated with the infiltration of follicular helper T cells, M0 macrophages, and activated dendritic cells. High LAPTM5 expression is linked to the upregulation of immunosuppressive TOX pathway genes, CD8^+^ T cell exhaustion, and shorter overall survival of LUSC patients, suggesting that LAPTM5 may modulate tumor infiltration, antitumor immunity, and survival outcomes in LUSC patients [17]. The abundance of tumor-infiltrating immune cells in lung adenocarcinoma (LUAD) and LUSC patients showed that B cells and myeloid dendritic cells (mDC) are independent prognostic factors in LUAD and LUSC patients, respectively. Enrichment analysis confirmed that genes highly associated with B cells or DC1 strongly correlated with immune activation in lung cancer patients, with LAPTM5 being highly related to DC1, suggesting its role in predicting the efficacy of immune checkpoint blockade (ICB) [18].

In bladder cancer (BCa), transcriptome analysis and immunohistochemical staining revealed significant upregulation of LAPTM. LAPTM5 knockdown triggers a cell-cycle arrest in the G0/G1 phase, resulting in a delayed BCa cell growth and inhibiting migration and invasion of BCa cells [10] (Fig. 2). In addition, LAPTM5 knockdown significantly reduced the phosphorylation of ERK1/2 and p38, key members of the MAPK family that regulates BCa tumorigenesis, suggesting that decreased LAPTM5 suppresses the proliferation and viability of BCa cells by inducing G0/G1 cell cycle arrest through inactivation ERK1/2 and p38 [10]. In testicular germ cell tumor (TGCT), analysis of GEO and TCGA databases identified LAPTM5 as a potential biomarker for diagnosis and prognosis, correlating with immune infiltration in TGCT [6].

However, a large-scale screening of tumor cell lines found that LAPTM5 mRNA levels were generally reduced in various cancer cell lines, including esophageal squamous (ESCC), prostate, undifferentiated thyroid, neck, hepatocellular carcinoma (HCC), breast, glioma, renal cell, endometrial, ovarian, bladder, gallbladder, small cell lung (SCLC), gastric, myeloma, non-small cell lung cancer (NSCLC), and pancreatic cancer [7]. Low LAPTM5 expression in ESCC and NSCLC patients was significantly associated with poor prognosis. Further analysis demonstrated that LAPTM5 overexpression induced lysosomal instability, autophagy impairment, and cathepsin D leakage, resulting in lysosomal cell death in ESCC and NSCLC cells [7]. LAPTM knockdown also affects autophagy pathways, potentially leading to non-apoptotic cell death rather than promoting cell proliferation. Thus, LAPTM5 downregulation suppresses tumorigenesis by inducing cell cycle arrest and affecting autophagic processes. These findings highlight the need for additional studies to validate the relevance of tumor cell lines as models for molecular expression and functional status of actual tumors.

### 4.4 Role of LAPTM5 in non-solid tumors

LAPTM5 also plays significant roles in non-solid tumors. In Waldenstrom's macroglobulinemia (WM), a proliferative disease of B-cell lymphoid tissue, whole-genome sequencing of germline DNA from 246 WM patients across four families revealed that the LAPTM5^c403t^ variants were prevalent in a larger number of family members. This suggests that LAPTM5 may be a candidate gene associated with familial WM, potentially linked to tumor clones [49]. Chromosome band 1p34 – 36 is a frequent rearrangement site in multiple cancers. In the human multiple myeloma cell line ODA, chromosomal translocation of t(1; 14) (p34; q32) occurs between the switch region of the immunoglobulin heavy chain (IgH) gene (Sμ) of 14q32 and the first intron site of the LAPTM5 gene at 1p34. This translocation results in a disruption of the LAPTM5 coding region and consequent loss of LAPTM5 expression in the ODA cell lines. Additionally, the remaining intact LAPTM5 alleles in ODA cells were silenced by DNA methylation of sequences near the first intronat a GC-rich *Eag*I site. These results suggest that the loss of LAPTM5 gene expression may be a critical event in the progression of multiple myeloma in humans [15].

## 5 Role of LAPTM5 in non-tumor immune-related contexts

Lysosomes play a crucial role in maintaining cellular homeostasis, and their dysregulation is linked to various human diseases. LAPTM5 is implicated in the molecular mechanisms of microglia-mediated synaptic phagocytosis, which is essential for the proper functioning of synaptic circuit [63]. These functional alterations of lysosome can contribute to pathologies related to CNS immunity, highlighting the significance of LAPTM5 beyond its roles in cancer. The involvement of LAPTM5 in these processes suggests that its dysregulation might drive non-tumor deficits, emphasizing the need for further research into its functions in non-cancerous conditions.

### 5.1 Role of LAPTM5 in neurological diseases

Currently, the role of LAPTM5 in the nervous system has not been extensively investigated. LAPTM5 acts as a key regulatory molecule in immune cells. Microglia, the resident immune cells of CNS, respond rapidly to injury and disease, playing a critical role in processes such as tissue inflammation and the removal of cellular debris [64]. Studies have suggested that microglia are implicated in synaptic remodeling and plasticity in the healthy brain [65]. Recent research evidence that disruption of microglia function leads to delayed maturation of synaptic circuits in the hippocampus [[65], [66], [67], [68]]. These findings prompt further investigations--whether LAPTM5 is involved in the molecular mechanisms of microglia-mediated synaptic phagocytosis and synaptic circuit function.

LAPTM5 is localized in the perinuclear region of cultured microglia, and its expression is upregulated during the early stages of granule cell death in cerebellar cell cultures, as indicated by the colocalization with the lysosomal marker ED1 antigen [63]. *In vivo*, LAPTM5 increased upon retinal nerve injury, suggesting its involvement in the kinetics of lysosomal membranes associated with microglial activation [63]. In a gene expression profiling analysis of a sciatic nerve chronic constriction injury (CCI), a significantly higher expression of LAPTM5 out of DEGs was identified in the dorsal horn of the spinal cord, and the PPI network analysis revealed LAPTM5 as a key node with a high degree of connectivity [69]. This suggests that LAPTM5 may be a neuroinflammation-related gene that affects neuropathic pain behavior post-CCI. However, the specific role of LAPTM5 in CCI and other CNS disorders needs to be further verified by extensive experiments.

Microglia play an important role in neurodegenerative diseases, with abnormal activation affecting processes such as phagocytic activity, synaptic pruning, and lipid metabolism in Alzheimer's disease (AD) [[70], [71], [72], [73], [74]]. An AD genome-wide association analysis (GWAS) study identified LAPTM5 as a risk gene associated with AD [75]. In a transgenic mouse model of Aβ deposition, LAPTM5 transcripts were significantly increased in the presence of amyloid plaques, resembling the increased number of LAPTM5 transcripts and increased microglial numbers [75]. Given that genetic variation in microglial response to amyloid deposition is a key determinant of AD risk, the identification of the LAPTM5 gene may aid in predicting the risk of developing AD [75]. In addition, AD is a multifactorial neurodegenerative disease, influenced by genetic and environmental risk factors, such as APOE4, sex, and diet, etc. Studies have shown that a high-fat diet (HFD) has significantly increased amyloid plaques in both male and female APP/E4 mice, with RNA sequencing and weighted gene co-expression network analysis showing a positive correlation of the LAPTM5-associated immune response network with the phenotype of female APP/E4-HFD mice [76]. Moreover, a gene expression profiling of idiopathic Parkinson's disease (IPD) revealed LAPTM5 as a hub node of the IPD regulatory network, suggesting its potential as a diagnostic and therapeutic target [77]. In dominant intermediate Charcot-Marie-Tooth (DI-CMT) neuropathy, haplotype analysis of two unrelated families unveiled LAPTM5 as a pathogenic mutation [78]. These findings underscore the importance of LAPTM5 in the nervous system, warranting further studies to elucidate its functions.

### 5.2 Role of LAPTM5 in the osteoarticular system

In addition to the roles of LAPTM5 in immune cells, tumors, and the nervous system, LAPTM5 also has a significant implication for the bone-joint system. DNA microarray analysis revealed elevated LAPTM5 expression in pigmented villonodular synovitis (PVNS) [79]. Additionally, silencing LAPTM5 expression in the mouse bone marrow stromal cell line (ST2) promotes the expression of the TNF superfamily member 11 (TNFSF11), which in turn promotes osteoclast differentiation and activation [[80], [81], [82]]. In this context, investigations into the potential implications of LAPTM2 in the osteoarticular system are necessarily needed in the future research.

## 6 Conclusions and future prospectives

LAPTM5 has emerged as a multifaceted player in the regulation of immune responses and immune-related biology. Its involvement spans various immune cells, including B cells, T cells, macrophages, and dendritic cells, indicating the potential implications of LAPTM5 in the development of various immune system-related diseases. Specifically, in the nervous system, the abnormal expression of LAPTM5 in microglia (immune cell in the nervous system) may be linked to the development of microglia-related diseases like AD and age-related neurodegeneration. LAPTM5's role in regulating microglial function and synaptic phagocytosis underscores its importance in maintaining CNS health. In tumors, immune infiltration is significantly associated with the tumorigenesis, substantially affecting the treatment and prognosis of patients. Functionally, the effect of LAPTM5 on tumor biology appears in several aspects: 1) Immune cell modulation: Upregulation of LAPTM5 in immune cells (such as T cells, B cells, macrophages, dendritic cells) can lead to decreased cell membrane levels of TCR or BCR, impair antigen presentation, and affect the immune infiltration. This contributes to immune escape, and promotes tumor development. 2) Cell death pathways: In tumor parenchymal cells, LAPTM5 expression is critical for inducing non-apoptotic cell death. Downregulation of LAPTM5 disrupts this cell death pathway in the tumor parenchyma, subsequently promoting tumor development. 3) Cell cycle regulation: LAPTM5 is also involved in the cell cycle, with its elevated expression of LAPTM5 in solid tumors rendering its inability to block in G0/G1 stage. The abnormal expression of LAPTM5 in non-solid tumors causes impaired blood cell development and abnormal proliferation. This review enlarges the understanding of LAPTM5 in the tumorigenesis and other immune-related disorders.

Nevertheless, the specific functions of LAPTM5 warrants further in-depth exploration. The Future research should focus on: 1) Elucidating LAPTM5 mechanisms: Further studies are needed to dissect the molecular mechanisms by which LAPTM5 influences immune cell function and tumor progression. Understanding these mechanisms will help in identifying its role in disease pathology. 2) Diagnostic and therapeutic applications: Developing LAPTM5-based diagnostic and therapeutic strategies could provide new avenues for managing tumors and immune-related disorders. Investigations into LAPTM5's potential as a biomarker and therapeutic target are crucial. 3) Exploring non-tumor contexts: More research is needed to explore LAPTM5's role in non-tumor contexts, particularly in neurological and osteoarticular systems, to fully understand its broader biological impact.

## Declarations ethics approval and consent to participate

Not applicable.

## Consent for publication

Not applicable.

## Funding

This work was financially supported by 10.13039/501100001809National Natural Science Foundation of China (No. 82060016) and Zunyi City Joint Fund Project (Zuncity Kehe HZ Zi (2021)-94).

## Authors' contributions

Study concept and design: SXL. Writing the paper: PPS, MML and PS. Revising the paper: JBY and SXL. All authors read and approved the final manuscript.

## Data availability statement

No data was used for the research described in the article.

## CRediT authorship contribution statement

**Peng-Peng Sun:** Writing – original draft. **Shi-Xia Liao:** Supervision, Conceptualization. **Peng Sang:** Resources. **Mao-Mao Liu:** Writing – review & editing. **Ji-Bin Yang:** Writing – original draft, Resources.

## Declaration of competing interest

The authors declare the following financial interests/personal relationships which may be considered as potential competing interests: Shi-Xia Liao reports financial support and writing assistance were provided by Zunyi City Joint Fund Project. If there are other authors, they declare that they have no known competing financial interests or personal relationships that could have appeared to influence the work reported in this paper.

## References

[1] Pak Y. (2006). Transport of LAPTM5 to lysosomes requires association with the ubiquitin ligase Nedd4, but not LAPTM5 ubiquitination. J. Cell Biol..

[2] Adra C.N. (1996). LAPTM5: a novel lysosomal-associated multispanning membrane protein preferentially expressed in hematopoietic cells. Genomics.

[3] Milkereit R., Rotin D. (2011). A role for the ubiquitin ligase Nedd4 in membrane sorting of LAPTM4 proteins. PLoS One.

[4] Wang Y. (2022). LAPTM5 mediates immature B cell apoptosis and B cell tolerance by regulating the WWP2-PTEN-AKT pathway. Proc Natl Acad Sci U S A.

[5] Berberich A. (2020). LAPTM5-CD40 crosstalk in glioblastoma invasion and temozolomide resistance. Front. Oncol..

[6] Li X. (2021). LAPTM5 plays a key role in the diagnosis and prognosis of testicular germ cell tumors. Int J Genomics.

[7] Nuylan M. (2016). Down-regulation of LAPTM5 in human cancer cells. Oncotarget.

[8] Sui Y., Lu K., Fu L. (2021). Prediction and analysis of novel key genes ITGAX, LAPTM5, SERPINE1 in clear cell renal cell carcinoma through bioinformatics analysis. PeerJ.

[9] Jun D.Y. (2017). Ectopic overexpression of LAPTM5 results in lysosomal targeting and induces Mcl-1 down-regulation, Bak activation, and mitochondria-dependent apoptosis in human HeLa cells. PLoS One.

[10] Chen L. (2017). Downregulation of LAPTM5 suppresses cell proliferation and viability inducing cell cycle arrest at G0/G1 phase of bladder cancer cells. Int. J. Oncol..

[11] Meng Q. (2022). Spine-specific downregulation of LAPTM5 expression promotes the progression and spinal metastasis of estrogen receptor-positive breast cancer by activating glutamine-dependent mTOR signaling. Int. J. Oncol..

[12] Inoue J. (2009). Lysosomal-associated protein multispanning transmembrane 5 gene (LAPTM5) is associated with spontaneous regression of neuroblastomas. PLoS One.

[13] Seimiya M. (2003). Stage-specific expression of Clast6/E3/LAPTM5 during B cell differentiation: elevated expression in human B lymphomas. Int. J. Oncol..

[14] Li B., Shi X.D. (2022). Key prognostic value of lysosomal protein transmembrane 5 in kidney renal clear cell carcinoma. Int. J. Gen. Med..

[15] Hayami Y. (2003). Inactivation of the E3/LAPTm5 gene by chromosomal rearrangement and DNA methylation in human multiple myeloma. Leukemia.

[16] Tu J. (2021). Prognostic and predictive value of a mRNA signature in peripheral T-cell lymphomas: a mRNA expression analysis. J. Cell Mol. Med..

[17] Zhang T. (2021). Four hub genes regulate tumor infiltration by immune cells, antitumor immunity in the tumor microenvironment, and survival outcomes in lung squamous cell carcinoma patients. Aging (Albany NY).

[18] Liu X. (2021). The prognosis and immune checkpoint blockade efficacy prediction of tumor-infiltrating immune cells in lung cancer. Front. Cell Dev. Biol..

[19] Cortese R. (2008). Correlative gene expression and DNA methylation profiling in lung development nominate new biomarkers in lung cancer. Int. J. Biochem. Cell Biol..

[20] INVALID CITATION !!! .

[21] Ouchida R. (2008). A lysosomal protein negatively regulates surface T cell antigen receptor expression by promoting CD3zeta-chain degradation. Immunity.

[22] Ouchida R., Kurosaki T., Wang J.Y. (2010). A role for lysosomal-associated protein transmembrane 5 in the negative regulation of surface B cell receptor levels and B cell activation. J. Immunol..

[23] Liu H. (2000). On the dynamics of TCR:CD3 complex cell surface expression and downmodulation. Immunity.

[24] Carrasco Y.R., Navarro M.N., Toribio M.L. (2003). A role for the cytoplasmic tail of the pre-T cell receptor (TCR) alpha chain in promoting constitutive internalization and degradation of the pre-TCR. J. Biol. Chem..

[25] Dietrich J. (2002). Ligand-induced TCR down-regulation is not dependent on constitutive TCR cycling. J. Immunol..

[26] San José E., Alarcón B. (1999). Receptor engagement transiently diverts the T cell receptor heterodimer from a constitutive degradation pathway. J. Biol. Chem..

[27] Geisler C. (2004). TCR trafficking in resting and stimulated T cells. Crit. Rev. Immunol..

[28] Friend S.F. (2013). SLAP deficiency increases TCR avidity leading to altered repertoire and negative selection of cognate antigen-specific CD8+ T cells. Immunol. Res..

[29] Dragone L.L. (2009). SLAP, a regulator of immunoreceptor ubiquitination, signaling, and trafficking. Immunol. Rev..

[30] Myers M.D. (2006). Src-like adaptor protein regulates TCR expression on thymocytes by linking the ubiquitin ligase c-Cbl to the TCR complex. Nat. Immunol..

[31] Naramura M. (2002). c-Cbl and Cbl-b regulate T cell responsiveness by promoting ligand-induced TCR down-modulation. Nat. Immunol..

[32] Thien C.B. (2005). Loss of c-Cbl RING finger function results in high-intensity TCR signaling and thymic deletion. Embo j.

[33] Kawai Y. (2014). LAPTM5 promotes lysosomal degradation of intracellular CD3ζ but not of cell surface CD3ζ. Immunol. Cell Biol..

[34] Zhu L., Chang C.H., Dunnick W. (2011). Excessive amounts of mu heavy chain block B-cell development. Int. Immunol..

[35] Chen J., Alt F.W. (1993). Gene rearrangement and B-cell development. Curr. Opin. Immunol..

[36] Mårtensson I.L. (2002). The pre-B cell receptor and its role in proliferation and Ig heavy chain allelic exclusion. Semin. Immunol..

[37] Eswaran J. (2015). The pre-B-cell receptor checkpoint in acute lymphoblastic leukaemia. Leukemia.

[38] Kawano Y. (2012). A novel mechanism for the autonomous termination of pre-B cell receptor expression via induction of lysosome-associated protein transmembrane 5. Mol. Cell Biol..

[39] Glowacka W.K. (2012). LAPTM5 protein is a positive regulator of proinflammatory signaling pathways in macrophages. J. Biol. Chem..

[40] Wang Y. (2022). HIV-1 Vif suppresses antiviral immunity by targeting STING. Cell. Mol. Immunol..

[41] Jäger S. (2011). Vif hijacks CBF-β to degrade APOBEC3G and promote HIV-1 infection. Nature.

[42] Sauter D. (2009). Tetherin-driven adaptation of Vpu and Nef function and the evolution of pandemic and nonpandemic HIV-1 strains. Cell Host Microbe.

[43] Neil S.J., Zang T., Bieniasz P.D. (2008). Tetherin inhibits retrovirus release and is antagonized by HIV-1 Vpu. Nature.

[44] Zhao L. (2021). Vpr counteracts the restriction of LAPTM5 to promote HIV-1 infection in macrophages. Nat. Commun..

[45] Angénieux C. (2012). Lysosomal-associated transmembrane protein 5 (LAPTM5) is a molecular partner of CD1e. PLoS One.

[46] Yang X. (2022). LCDR regulates the integrity of lysosomal membrane by hnRNP K-stabilized LAPTM5 transcript and promotes cell survival. Proc Natl Acad Sci U S A.

[47] Ishihara T. (2011). HECT-type ubiquitin ligase ITCH targets lysosomal-associated protein multispanning transmembrane 5 (LAPTM5) and prevents LAPTM5-mediated cell death. J. Biol. Chem..

[48] Gao Y., Chen Q., Yue W. (2019). 30P - LAPTM5 protein can regulate TGF-β mediated MAPK and smad signaling pathways in ovarian cancer cell. Ann. Oncol..

[49] Roccaro A.M. (2016). Exome sequencing reveals recurrent germ line variants in patients with familial Waldenström macroglobulinemia. Blood.

[50] Gn M.H. (2020). Aberrant expression of RSK1 characterizes high-grade gliomas with immune infiltration. Mol. Oncol..

[51] Xiao X. (2022). Old wine in new bottles: kaempferol is a promising agent for treating the trilogy of liver diseases. Pharmacol. Res..

[52] Cao N. (2020). Application of curved ablation in liver cancer with special morphology or location: report of two cases. World J Clin Cases.

[53] Ginès P. (2022). Population screening for liver fibrosis: toward early diagnosis and intervention for chronic liver diseases. Hepatology.

[54] Mello T. (2008). Alcohol induced hepatic fibrosis: role of acetaldehyde. Mol. Aspect. Med..

[55] Sánchez-Jiménez B.A. (2018). Both alcoholic and non-alcoholic steatohepatitis association with cardiovascular risk and liver fibrosis. Alcohol.

[56] Schuppan D. (2018). Liver fibrosis: direct antifibrotic agents and targeted therapies. Matrix Biol..

[57] Messner C.J. (2021). Single cell gene expression analysis in a 3D microtissue liver model reveals cell type-specific responses to pro-fibrotic TGF-β1 stimulation. Int. J. Mol. Sci..

[58] Liu X. (2020). Primary alcohol-activated human and mouse hepatic stellate cells share similarities in gene-expression profiles. Hepatol Commun.

[59] Yin X. (2020). Assessment for prognostic value of differentially expressed genes in immune microenvironment of clear cell renal cell carcinoma. Am J Transl Res.

[60] Turner M.C. (2020). Outdoor air pollution and cancer: an overview of the current evidence and public health recommendations. CA Cancer J Clin.

[61] Hamann H.A. (2018). Multilevel opportunities to address lung cancer stigma across the cancer control continuum. J. Thorac. Oncol..

[62] Jiang B. (2022). Lysosomal protein transmembrane 5 promotes lung-specific metastasis by regulating BMPR1A lysosomal degradation. Nat. Commun..

[63] Origasa M. (2001). Activation of a novel microglial gene encoding a lysosomal membrane protein in response to neuronal apoptosis. Brain Res Mol Brain Res.

[64] Schafer D.P. (2012). Microglia sculpt postnatal neural circuits in an activity and complement-dependent manner. Neuron.

[65] Paolicelli R.C. (2011). Synaptic pruning by microglia is necessary for normal brain development. Science.

[66] Wang C. (2021). Selective removal of astrocytic APOE4 strongly protects against tau-mediated neurodegeneration and decreases synaptic phagocytosis by microglia. Neuron.

[67] Favuzzi E. (2021). GABA-receptive microglia selectively sculpt developing inhibitory circuits. Cell.

[68] Hong S. (2016). Complement and microglia mediate early synapse loss in Alzheimer mouse models. Science.

[69] Du H. (2018). Analyses of gene expression profiles in the rat dorsal horn of the spinal cord using RNA sequencing in chronic constriction injury rats. J. Neuroinflammation.

[70] Keren-Shaul H. (2017). A unique microglia type associated with restricting development of Alzheimer's disease. Cell.

[71] Lui H. (2016). Progranulin deficiency promotes circuit-specific synaptic pruning by microglia via complement activation. Cell.

[72] Shi Y. (2019). Microglia drive APOE-dependent neurodegeneration in a tauopathy mouse model. J. Exp. Med..

[73] Yousef H. (2019). Aged blood impairs hippocampal neural precursor activity and activates microglia via brain endothelial cell VCAM1. Nat Med.

[74] Nguyen P.T. (2020). Microglial remodeling of the extracellular matrix promotes synapse plasticity. Cell.

[75] Salih D.A. (2019). Genetic variability in response to amyloid beta deposition influences Alzheimer's disease risk. Brain Commun.

[76] Nam K.N. (2018). Integrated approach reveals diet, APOE genotype and sex affect immune response in APP mice. Biochim. Biophys. Acta, Mol. Basis Dis..

[77] Cui S. (2015). Gene expression profiling analysis of locus coeruleus in idiopathic Parkinson's disease by bioinformatics. Neurol. Sci..

[78] Jordanova A. (2003). Dominant intermediate Charcot-Marie-Tooth type C maps to chromosome 1p34-p35. Am. J. Hum. Genet..

[79] Finis K. (2006). Analysis of pigmented villonodular synovitis with genome-wide complementary DNA microarray and tissue array technology reveals insight into potential novel therapeutic approaches. Arthritis Rheum..

[80] Lindman B.R. (2016). Calcific aortic stenosis. Nat. Rev. Dis. Prim..

[81] Luo J. (2016). LGR4 is a receptor for RANKL and negatively regulates osteoclast differentiation and bone resorption. Nat Med.

[82] Geng Y.M. (2019). LAPTM5 is transactivated by RUNX2 and involved in RANKL trafficking in osteoblastic cells. Mol. Med. Rep..

